# Efficacy and Safety of *Lythrum salicaria* Extract on Body Fat Reduction in Adults with a BMI of 23–30 kg/m^2^: A Randomized, Double-Blind, Placebo-Controlled Trial

**DOI:** 10.3390/nu18203342

**Published:** 2026-10-12

**Authors:** Bong-Jin Shin, Hye-Jin Park, Yea-Jin Park, Hyo-Jin An, Su Shin, Yun-Yeop Cha, In Heo

**Affiliations:** 1 Department of Korean Medicine, The Graduate School, Pusan National University, Yangsan 50612, Republic of Korea; shinbj0831@gmail.com; 2 Department of Korean Medicine Rehabilitation, Pusan National University Korean Medicine Hospital, 20 Geumo-ro, Mulgeum-eup, Yangsan 50612, Republic of Korea; 3 Department of Rehabilitation Medicine of Korean Medicine, Se-Myung University Chungju Korean Medicine Hospital, 63 Sangbang 4-gil, Chungju 27429, Republic of Korea; 4 Department of Rehabilitation Medicine of Korean Medicine, College of Korean Medicine, Sangji University, 80 Sangjidae-gil, Wonju 26338, Republic of Korea; 5 College of Pharmacy and Institute of Integrated Pharmaceutical Sciences, Kyung Hee University, 26 Kyungheedae-ro, Dongdaemun-gu, Seoul 02447, Republic of Korea; 6 Department of Precision Medicine, Graduate School, Kyung Hee University, Seoul 02447, Republic of Korea; 7 Department of Korean Medical Science, School of Korean Medicine, Pusan National University, Yangsan 50612, Republic of Korea; 8 The School of Korean Medicine, Pusan National University, Yangsan 50612, Republic of Korea

**Keywords:** *Lythrum salicaria*, body fat, dual-energy X-ray absorptiometry, BMI, adiponectin, randomized controlled trial

## Abstract

**Background/Objectives**: Plant-derived ingredients have attracted interest as adjuncts for reducing body fat, but clinical evidence for *Lythrum salicaria* extract alone remains limited. We evaluated its effects on body fat and its safety in adults with a body mass index (BMI) of 23–30 kg/m^2^. **Methods**: Ninety adults aged 19–75 years participated in a single-center, randomized, double-blind, placebo-controlled trial. Participants received either 500 mg/day of *L. salicaria* extract powder (corresponding to approximately 400 mg/day of plant-derived extract solids) or placebo for 12 weeks. The primary endpoint was the change in body fat mass from baseline to day 84, assessed by dual-energy X-ray absorptiometry (DXA). Efficacy was evaluated primarily in the per-protocol (PP) population, with supporting intention-to-treat (ITT) analyses. Anthropometric measures, other DXA outcomes, and blood markers were assessed as secondary endpoints. **Results**: The PP population included 84 participants. Baseline-adjusted differences in body fat mass change, expressed as extract minus placebo, were −2.01 kg (95% confidence interval [CI], −2.51 to −1.52; *p* < 0.001) in the PP population and −2.04 kg (95% CI, −2.66 to −1.42; *p* < 0.001) in the ITT population. Exploratory secondary analyses favored the extract for BMI, body weight, body fat percentage, and adiponectin. No serious adverse events occurred. **Conclusions**: Supplementation with *L. salicaria* extract powder for 12 weeks was associated with a reduction in DXA-measured body fat mass compared with placebo. Larger trials with longer follow-up are needed to confirm the findings and investigate the mechanisms involved.

## 1. Introduction

Obesity, an excess of accumulated body fat, raises the risk of type 2 diabetes, dyslipidemia, hypertension, cardiovascular disease, and sleep-disordered breathing [1,2]. For screening, body mass index (BMI) remains widely used because it is readily calculated in both clinical practice and population surveys. The World Health Organization (WHO) sets the adult BMI thresholds for overweight and obesity at BMI 25.0 kg/m^2^ and ≥30 kg/m^2^, respectively [3]. At a given BMI, some Asian populations generally have a higher body fat percentage and greater metabolic risk than European populations [4]. The WHO Expert Consultation therefore identified 23 kg/m^2^ as the threshold for increased risk in Asian populations [4]. Korean guidelines classify a BMI of 23.0–24.9 kg/m^2^ as overweight, or pre-obesity, and a BMI of ≥25.0 as obesity [5].

Overweight and obesity have become increasingly prevalent. Worldwide, prevalence rose from 30.5% to 46.7% in women and from 27.6% to 43.4% in men between 1990 and 2021, and the 2050 projections reach 60.3% and 57.4% [6]. In Korea, BMI-defined obesity affected 38.4% of adults in 2022 [7].

Reliance on BMI has limitations when evaluating interventions intended to reduce body fat. Most weight-loss programs gauge success by weight or BMI, neither of which distinguishes fat loss from muscle loss [8]. Assessment of body composition is therefore needed to interpret these changes. Dual-energy X-ray absorptiometry (DXA) estimates fat, lean, and bone masses separately, allowing the contribution of each component to be examined [9].

First-line obesity management includes dietary modification, behavioral intervention, and increased physical activity, with pharmacotherapy or bariatric surgery reserved for selected individuals [10]. Because obesity requires lifelong management, interventions must demonstrate not only efficacy but also long-term safety, sustainability, and feasibility in routine practice [10]. These considerations have increased interest in plant-derived compounds, which may influence fat absorption, energy metabolism, adipocyte differentiation, and lipid metabolism [11,12].

*Lythrum salicaria* L. is a perennial in the Lythraceae family. Its heteropolysaccharides and polyphenols—in particular, C-glucosidic ellagitannins and C-glucosidic flavonoids—show antioxidant, anti-inflammatory, and antihyperglycemic activities [13]. Ellagitannins are poorly absorbed, but hydrolysis can release ellagic acid, which gut microbiota can then convert to urolithins. These metabolites may contribute to the systemic effects of ellagitannins [14]. *L. salicaria* was identified as a promising candidate for anti-obesity research through a native-plant screening program conducted by the Gangwon-do Agricultural Research and Extension Services [15]. This development background provided the rationale for further investigation of *L. salicaria*. Subsequent preclinical work found that an aqueous extract inhibited the differentiation of 3T3-L1 cells into white adipocytes and attenuated the increase in body weight associated with high-fat feeding in mice [16]. These effects were associated with reduced adipogenesis and lipogenesis, enhanced fatty acid oxidation, and activation of AMP-activated protein kinase (AMPK) [16].

Clinical evidence for *L. salicaria* remains limited. In an 8-week preliminary randomized trial, adults with a BMI of 23.0–29.9 kg/m^2^ received a combined ethanol extract of *L. salicaria* and *Mukdenia rossii* (syn. *Aceriphyllum rossii*). Compared with placebo, the intervention did not significantly affect visceral or subcutaneous fat area measured using abdominal computed tomography (CT), limb or trunk circumference, or abdominal temperature assessed using digital infrared thermal imaging [17]. However, because the intervention combined two herbal extracts, the independent contribution of *L. salicaria* could not be determined. We therefore evaluated *L. salicaria* extract alone in adults aged 19–75 years with a BMI of 23.0–30.0 kg/m^2^. DXA-measured body fat mass was the primary endpoint, with anthropometric measurements, other body-composition measures, and blood markers providing additional information on its effects.

## 2. Materials and Methods

### 2.1. Study Design and Ethical Considerations

We conducted this single-center trial at Pusan National University Korean Medicine Hospital using a randomized, double-blind comparison of *L. salicaria* and placebo. The study had two parallel arms with 1:1 allocation and was designed to assess superiority. Reporting follows Consolidated Standards of Reporting Trials (CONSORT) 2025 [18]. The study design had been described previously and published as a separate protocol paper [19] (https://doi.org/10.15429/jkomor.2024.24.1.87). The hospital’s institutional review board (IRB) approved the study (PNUKHIRB 2022-08-002; approved 14 September 2022). We registered the trial with the Clinical Research Information Service (CRIS; KCT0008060; 2 January 2023); https://trialsearch.who.int/Trial2.aspx?TrialID=KCT0008060 (accessed on 9 October 2026)). There were no important changes to the protocol once the trial was underway. The first participant provided informed consent on 16 February 2023. Participant enrollment and randomization were conducted from 3 March to 21 August 2023, and the final follow-up assessment was completed on 9 November 2023. Patients and the public did not contribute to planning the trial, conducting it, or preparing its report.

### 2.2. Inclusion and Exclusion Criteria

Men and women who voluntarily provided written consent were eligible if their age was 19–75 years and their BMI was 23–30 kg/m^2^.

Exclusion criteria were: (1) severe cerebrovascular disease, cardiac disease, or malignancy within the previous 6 months; (2) uncontrolled hypertension; (3) diabetes with uncontrolled glycemia (fasting glucose ≥ 160 mg/dL); (4) treatment for hypo- or hyperthyroidism; (5) serum creatinine ≥ 2× the upper limit of normal at the study laboratory; (6) aspartate aminotransferase (AST) or alanine aminotransferase (ALT) ≥ 3× the upper limit of normal; (7) severe gastrointestinal symptoms such as heartburn, dyspepsia, or lactose intolerance; (8) use of any weight-altering medication within the previous month; (9) participation in a commercial weight-loss program within the previous 3 months; (10) participation in, or planned participation in, another clinical trial within the previous month; (11) alcohol abuse; (12) smoking cessation within the previous 3 months; (13) current or planned pregnancy or breastfeeding; (14) allergy to any study food component; and (15) any other condition deemed unsuitable by an investigator.

Reasons for withdrawal or suspension were: (1) breach of the eligibility criteria; (2) a request to withdraw; (3) an adverse event tied to the study product or otherwise likely to affect the trial; (4) compliance below 80% or intake that could not be verified; (5) major protocol deviation; (6) use of a drug or functional food able to sway the results; (7) more than five consecutive days off the product; or (8) other grounds at the investigators’ discretion.

### 2.3. Sample Size

We planned the sample size to detect a difference in body fat mass change between the extract and placebo groups. Because directly comparable clinical data on *L. salicaria* extract alone were limited, the sample size assumptions were based on investigator judgment. For sample-size planning, the investigators assumed a between-group difference of 1.5 kg over 12 weeks and a standard deviation (SD) of 2.38 kg. With a two-sided *α* of 0.05 and 80% power, the normal-approximation formula for comparing two independent means with equal allocation gives

$$n=\frac{2\sigma^{2}{\left(Z_{1-\alpha /2}+Z_{1-\beta }\right)}^{2}}{\delta^{2}}\approx \frac{2{\left(2.38\right)}^{2}{\left(1.96+0.8416\right)}^{2}}{{\left(1.5\right)}^{2}}\approx 39.52,$$

where *n* is the required sample size per arm, *σ* = 2.38 kg is the assumed common SD of body fat mass changes, *δ* = 1.5 kg is the target between-group difference, *β* = 0.20, and *Z_p_* denotes the *p*th quantile of the standard normal distribution. Rounding up yielded 40 evaluable participants per arm. We therefore set the total recruitment target at 90 participants, with 45 in each arm, to allow for 10% attrition.

### 2.4. Randomization and Blinding

We used block randomization to allocate eligible participants equally between the *L. salicaria* extract and control arms. An independent statistician generated the randomization sequence, prespecified the block size, and concealed the allocation schedule. Participants were assigned sequential study numbers and received the corresponding intervention according to the randomization sequence. The investigational products were identical in appearance, and treatment allocation remained concealed from both participants and investigators until completion of the trial. The randomization sequence was maintained in sequentially numbered, sealed, opaque envelopes by the study monitor or an independent third party.

### 2.5. Intervention

#### 2.5.1. Study Products and Manufacturing

Participants in the experimental group took one 450-mg hard capsule (166.7 mg of *L. salicaria* extract powder with 283.3 mg of lactose) three times a day, with water, in the morning, at midday, and in the evening for 84 days. The total daily intake comprised 1350 mg of capsule contents, including 500 mg of *L. salicaria* extract powder. The extract powder consisted of 80% plant-derived extract solids and 20% maltodextrin, corresponding to approximately 400 mg/day of plant-derived extract solids and 100 mg/day of maltodextrin. The control group maintained the same schedule with 450-mg placebo capsules containing 355 mg of lactose, 81 mg of Caramel Color III, and 14 mg of Caramel Color I. The caramel colorants were omitted from the placebo description in the published protocol [19]. These colorants were included in the placebo formulation from the start of the trial, and the formulation remained unchanged throughout the study.

The extract powder was prepared from the dried aerial parts of *Lythrum salicaria*. It was developed by BIOPORT KOREA Inc. (Busan, Republic of Korea) and manufactured under contract by Bolak Co., Ltd. (Hwaseong, Republic of Korea); the finished investigational products were manufactured by DayNbio Co., Ltd. (Jangheung, Republic of Korea). The extract powder lots used in the trial were F12210001, F12210002, and F12210003. The finished-product lot numbers were DB-112401 for the active product and DB-111801 for the placebo.

For each lot, 20 kg of dried plant material underwent an initial 3-h extraction at 100 °C using purified water, with a solvent-to-material ratio of 26:1. The extract was then passed through a 4-μm filter. The remaining plant material underwent a further 1-h extraction at 100 °C with a solvent-to-material ratio of 20:1, followed by filtration. After pooling, the filtrates underwent vacuum concentration at a temperature of at least 60 °C until the concentration reached at least 30 °Brix. The extract solids were mixed with maltodextrin at an 80:20 ratio, heat-treated at 85 °C for 40 min, and spray-dried at inlet and outlet temperatures of 180–190 °C and 85–95 °C, respectively. The mean yield of the spray-dried powder, including maltodextrin, was 18.0% relative to the dried plant material (range, 17.5–18.5%). The study products were stored at 1–30 °C and used within their 24-month shelf life.

#### 2.5.2. Chemical Characterization and Quality Control

The analysis was performed by the Busan Branch of the Korea Food Industry Association (Busan, Republic of Korea). Chemical characterization used orientin as the marker compound. Orientin was quantified by high-performance liquid chromatography using a CAPCELL PAK C18 UG80 column (250 × 4.6 mm, 5 μm) maintained at 30 °C, with ultraviolet detection at 345 nm. The mobile phases were acetonitrile (A) and water containing 1% phosphoric acid (B). The gradient program was as follows: 15.5% A at 0–5 min, increasing to 28% A at 25 min, returning to 15.5% A at 25.1 min, and maintained at 15.5% A until 30 min. The flow rate was 0.8 mL/min, and the injection volume was 20 μL. For sample preparation, approximately 100 mg of extract powder was mixed with 25 mL of distilled water, sonicated for 20 min, diluted to 50 mL with distilled water, and passed through a 0.45-μm syringe filter. An orientin stock standard solution (200 μg/mL) was prepared in 50% methanol and diluted with distilled water to prepare working standard solutions. Orientin content was calculated from the sample-to-standard peak-area ratio, accounting for dilution, sample mass, and standard purity. The orientin concentrations were 0.330, 0.329, and 0.335 mg/g in lots F12210001, F12210002, and F12210003, respectively, and fell within the specified range of 0.264–0.396 mg/g (0.33 mg/g, 80–120%).

Quality-control testing showed lead, cadmium, and arsenic concentrations of 0.17, 0.05, and 0.37–0.39 mg/kg, respectively. Total mercury was reported as 0.00 mg/kg after rounding of the measured concentration. All 117 pesticides tested using Korean Food Code methods were reported as not detected, corresponding to concentrations below the limit of quantification (LOQ) of 0.01 mg/kg for each pesticide, as confirmed by the testing laboratory. Results and the complete pesticide panel are provided in Table S1.

#### 2.5.3. Dose Justification and Adherence

The daily dose was selected based on 100 mg/kg/day, a dose evaluated in the preclinical mouse study [16]. A conversion factor of approximately 0.08, based on body surface area, was applied to translate the mouse dose to its human equivalent. The resulting dose was approximately 8 mg/kg/day, equivalent to 480 mg/day for an adult weighing 60 kg [20]. The dose was set at 500 mg/day of *L. salicaria* extract powder, corresponding to approximately 400 mg/day of plant-derived extract solids.

Returned intake diaries and unused study products were used to determine the number of doses taken. Adherence was expressed as a percentage by dividing this number by the prescribed number of doses and multiplying the result by 100. Any concomitant treatment, medication, or functional food was recorded in the case report form and was allowed only when required for safety reasons and when it seemed unlikely to interfere with the effects of the study product.

### 2.6. Visit Schedule and Assessments

Four visits were planned: screening (day −21 to 0), baseline (day 0), day 42 ± 7, and day 84 ± 7. At screening, participants consented and underwent eligibility checks, history, physical examination, vital signs, and laboratory tests. At baseline, those who qualified were randomized and given their product. On day 42 we resupplied the product and reviewed adherence, adverse events, and concomitant drugs. On day 84 we assessed efficacy and safety and again reviewed adverse events and concomitant drugs.

### 2.7. Efficacy Endpoints

For the primary endpoint, DXA measurements were used to calculate body fat mass at day 84 minus the baseline value. Secondary endpoints were BMI, body weight, waist-to-hip ratio (WHR), body fat percentage, lean mass, a lipid panel (total cholesterol, triglyceride, high-density lipoprotein cholesterol [HDL-C], and low-density lipoprotein cholesterol [LDL-C]), free fatty acid (FFA), high-sensitivity C-reactive protein (hs-CRP), adiponectin, and leptin.

DXA fat mass, fat percentage, and lean mass were measured at Pusan National University Yangsan Hospital at baseline and day 84 using a Horizon W DXA scanner (Hologic, Inc., Marlborough, MA, USA) with software version 13.6.0.5 and the whole-body analysis protocol. BMI, body weight, waist and hip circumference, and WHR were measured at baseline, day 42, and day 84, and blood markers at baseline and day 84. Participants fasted for at least 6 h before blood collection. The waist was measured at the level midway between the lowest rib’s lower margin and the iliac crest’s upper border. The hip measurement was taken around the widest part of the buttocks. Diet and activity were evaluated at baseline and day 84 using a 3-day dietary assessment and the International Physical Activity Questionnaire, respectively [21].

### 2.8. Safety Assessment

Safety metrics included adverse events, abnormal vital signs or medical history, and clinically meaningful shifts in blood chemistry. Laboratory tests included white blood cell count, hemoglobin, platelet count, glucose, creatinine, aspartate aminotransferase, and alanine aminotransferase. These were examined in every participant who took at least one dose after randomization. Adverse events were graded as mild, moderate, or severe. Causality was set based on predefined criteria; a laboratory or vital-sign abnormality counted as clinically significant when the investigator judged it relevant. Serious adverse events were to be reported to the sponsor and the IRB according to the protocol.

### 2.9. Analysis Sets and Statistical Analysis

#### 2.9.1. Analysis Populations

The published protocol specified the per-protocol (PP) population for the principal efficacy analysis, with the intention-to-treat (ITT) population providing supporting analyses [19]. This choice reflected the study’s focus on the body-fat response to the prescribed 12-week supplementation regimen among participants meeting the predefined adherence and protocol-compliance criteria. The ITT population included all randomized participants who took the assigned study product at least once. The PP population comprised participants in the ITT population who completed the week-12 visit, met the prespecified adherence criteria, including intake of at least 80% of the prescribed doses, and had no major protocol deviations.

#### 2.9.2. Multiple Imputation

In the ITT population, missing values were filled by multiple imputations [22]. A fully conditional specification (FCS) approach was adopted, with continuous variables imputed by predictive mean matching using five candidate donors. Imputation was performed jointly across treatment groups, with efficacy variables, safety variables, and dietary intake and physical activity variables imputed in separate blocks.

Treatment allocation was included as a predictor in every block. The efficacy block included baseline and week-12 DXA body fat mass, body fat percentage, lean mass, BMI, body weight, WHR, triglycerides, HDL-C, LDL-C, total cholesterol, FFA, hs-CRP, adiponectin, and leptin, together with week-6 BMI, body weight, and WHR. The laboratory safety block included baseline and week-12 white blood cell count, hemoglobin, platelet count, glucose, creatinine, AST, and ALT. The dietary intake and physical activity block included baseline and week-12 three-day total energy intake and physical activity. Baseline measurements were used as predictors; follow-up measurements were used as predictors and imputed where missing.

Twenty imputed datasets were generated under a missing-at-random (MAR) assumption, using 20 FCS iterations and a random seed of 2,000,000. Convergence was assessed by visual inspection of FCS iteration-history plots, which showed fluctuations within stable ranges without persistent directional trends. Missing week-12 measurements were imputed before calculating changes from baseline, and estimates from analyses of the imputed datasets were pooled using Rubin’s rules. Imputed week-12 body weight and DXA lean mass values were reviewed alongside observed baseline measurements to assess the plausibility of the resulting changes.

MAR was adopted as a working assumption conditional on treatment allocation and observed measurements. Because this assumption could not be verified from the observed data alone, sensitivity to alternative missing-data assumptions was assessed.

#### 2.9.3. Statistical Methods

We report means and SDs for continuous variables and counts and percentages for categorical variables. Within individual imputed datasets, descriptive SDs were calculated from the group-mean standard errors (SEs) using SD = SE × √*n*, rather than from pooled standard errors. In a post hoc descriptive analysis, individual changes in body fat mass among participants with paired baseline and week-12 DXA measurements were summarized using medians and interquartile ranges, together with the number and proportion of participants with any observed decrease.

The Shapiro–Wilk test assessed normality. For between-group comparisons, test selection considered normality, skewness, kurtosis, and the presence of extreme values identified using boxplots. Independent-samples *t* tests were used for parametric comparisons. Welch’s *t* test was used for parametric between-group comparisons when the equal-variance assumption was not met. For rank-based comparisons, Mann–Whitney U tests were used in the PP population. For rank-based comparisons in the ITT population, values were rank-transformed separately within each of the 20 imputed datasets. Independent-samples *t* tests were applied to the rank-transformed values, and the between-group mean-rank differences and their variances were pooled using Rubin’s rules in SPSS Statistics 26.0 (IBM Corp., Armonk, NY, USA). Detailed distributional diagnostics and assessments of homogeneity of variance are provided in Supplementary Table S2. Depending on the data, categorical variables were assessed with either Fisher’s exact test or the chi-square test.

Between-group efficacy was assessed by comparing changes from baseline. For each endpoint in the PP population, analysis of covariance (ANCOVA) modeled the change from baseline as a function of treatment group and the corresponding baseline value. Additional analyses applied the same baseline-adjusted ANCOVA model to the primary endpoint in the ITT population. The baseline-adjusted ANCOVA models were assessed for residual normality, homogeneity of residual variances, and homogeneity of regression slopes. We used two-sided tests and considered *p* < 0.05 statistically significant. The main analyses were performed using SPSS Statistics 26.0, and the additional imputation-model sensitivity analyses were performed using R version 4.3.3 (R Foundation for Statistical Computing, Vienna, Austria). No adjustment for multiple comparisons was applied.

#### 2.9.4. Additional and Sensitivity Analyses

An additional analysis included age as a covariate in the baseline-adjusted PP model for the primary endpoint. All other analyses described in this section were conducted post hoc.

The influence of extreme observed changes in DXA-measured body fat mass was assessed in the PP population. Extreme values were defined using group-specific boxplots as changes below the first quartile minus 3 interquartile ranges (IQRs) or above the third quartile plus 3 IQRs. The baseline-adjusted ANCOVA was repeated after excluding participants with extreme values while retaining mild outliers. The original PP analysis remained the principal efficacy analysis.

Sensitivity to missing-data assumptions for the primary endpoint was assessed using a scenario unfavorable to the extract group. Missing day-84 body fat mass values were imputed using the greatest observed percentage decrease within the control group and the greatest observed percentage increase within the extract group, respectively. Between-group differences in changes from baseline were assessed using both the independent-samples *t* test and the Mann–Whitney U test.

For body weight and DXA lean mass, observed week-12 measurements were retained, and alternative values were assigned to the six participants with missing follow-up data. Assumptions were based on the extreme percentage changes observed among the 84 participants with paired measurements, pooling both treatment groups. For body weight, changes of −8.53% and +4.71% were applied to baseline values in the control and extract groups, respectively. For lean mass, the corresponding changes were +7.33% and −8.78%. Between-group differences in change were assessed using independent-samples *t* tests.

Sensitivity to imputation-model specification was examined for body weight and DXA lean mass. Twenty datasets were generated for each outcome using outcome-specific predictive mean matching with five candidate donors (seed 2,000,000). Imputation models included treatment allocation, the corresponding baseline value, age, and sex. The week-12 body-weight model additionally included week-6 body weight; missing week-6 values in two participants were imputed before week-12 values. Observed measurements were retained. Within each completed dataset, unadjusted between-group differences in mean change were estimated using treatment-only linear models with HC3 heteroscedasticity-consistent standard errors. Effect estimates and variances were pooled using Rubin’s rules with Barnard–Rubin degrees of freedom. The same treatment-comparison model was applied to complete cases.

For outcomes with identified model-assumption concerns, ANCOVA analyses evaluated changes in natural-log-transformed measurements, defined as ln(week-12 value) minus ln(baseline value), with baseline adjustment. Model assumptions were reassessed using formal diagnostic tests and graphical examination of the baseline–change relationships and residual-versus-fitted plots (Figure S1). Conventional model-based confidence intervals (CIs) were reported for these additional ANCOVA analyses.

## 3. Results

### 3.1. Participant Flow and Analysis Sets

**Figure 1:**
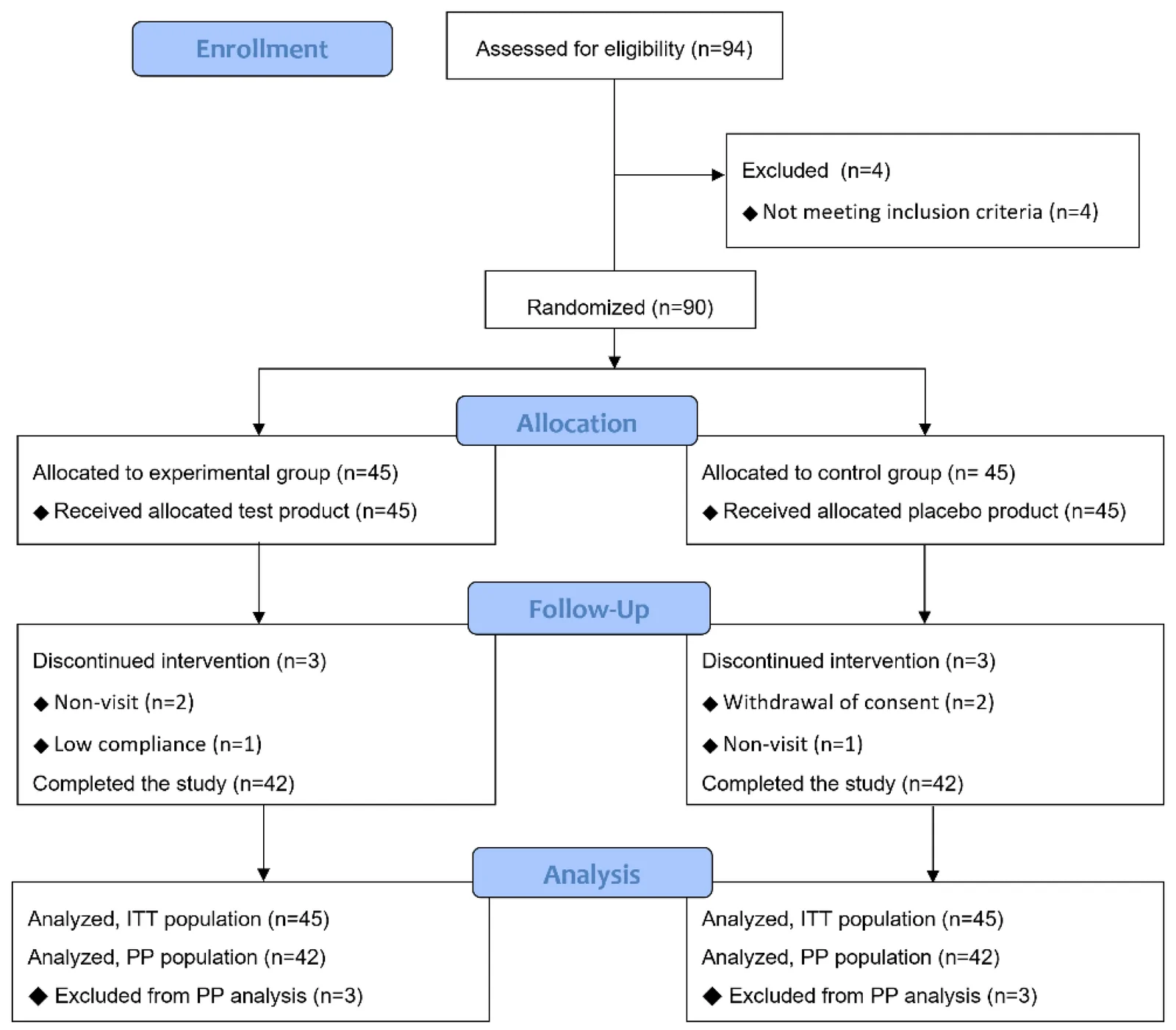
**Consolidated Standards of Reporting Trials** flow diagram. ITT, intention-to-treat; PP, per-protocol.

We screened 94 participants and randomly allocated the 90 who were eligible to the *L. salicaria* extract and control groups, with 45 participants in each group. Six participants left the study during the trial: two missed the visits, one had low compliance in the extract group, another two withdrew consent, and one missed a visit in the control group. That left 84 participants, with 42 per group in the PP population and 90 in the ITT population (45 per group); safety covered all 90. Participant withdrawals were not considered related to the study procedures or to the efficacy or safety endpoints, and no major protocol deviation occurred (Figure 1). All six participants excluded from the PP population did not attend the week-12 visit. For the primary and all secondary efficacy endpoints, the complete-case populations, comprising participants with observed baseline and week-12 measurements for the respective endpoints, were identical to the PP population (84 participants; 42 per group). Among the six participants with missing week-12 measurements, baseline body weight and DXA measurements were available for all six, and week-6 body weight was available for four.

### 3.2. Baseline Characteristics

Baseline comparisons in the ITT population yielded nonsignificant results for age, height, weight, body temperature, systolic or diastolic blood pressure, pulse, sex, smoking status, and alcohol use. Within the PP population, only the age comparison reached statistical significance (*p* = 0.038; Table 1).

### 3.3. Primary Efficacy Endpoint

#### 3.3.1. Changes in Body Fat Mass

**Figure 2:**
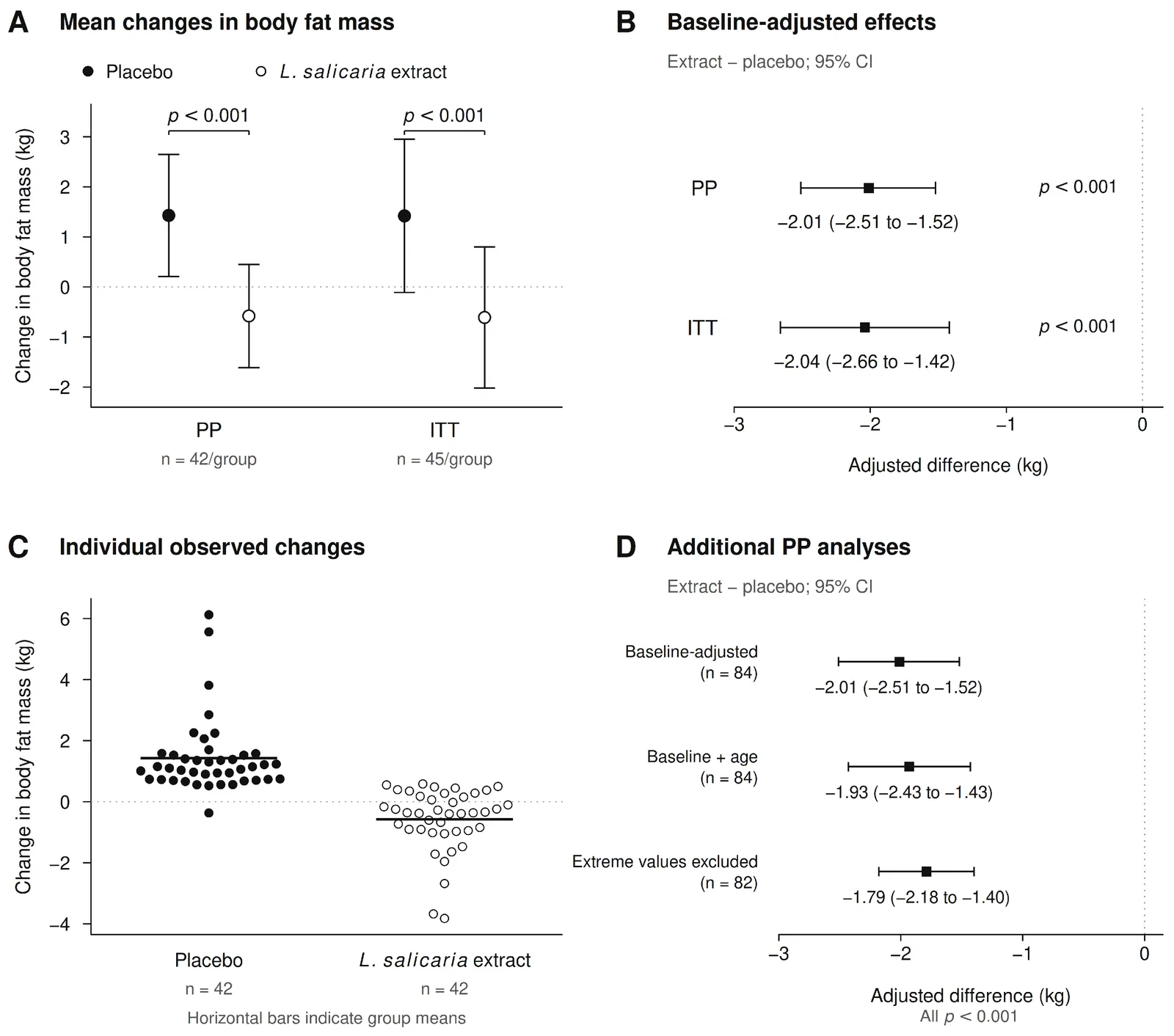
Changes in DXA-measured body fat mass over 12 weeks. (**A**) Mean changes from baseline to week 12 in the PP and ITT populations; error bars represent SDs. (**B**) Baseline-adjusted between-group differences in change (extract minus placebo), with 95% confidence intervals, in the PP and ITT populations. (**C**) Individual observed changes for all 84 participants with paired measurements (42 per group); horizontal bars indicate group means. Points are horizontally displaced to avoid overlap; imputed values are not shown. (**D**) PP analyses adjusted for baseline alone or baseline and age (42 per group), and baseline-adjusted analysis excluding two extreme placebo-group changes outside the group-specific 3-IQR fences (40 placebo, 42 extract). Filled and open circles in (**A**,**C**) represent placebo and *L. salicaria* extract, respectively. Squares and horizontal bars in (**B**,**D**) indicate adjusted differences (extract minus placebo) and 95% CIs. Dotted reference lines indicate zero change in (**A**,**C**) and no between-group difference in (**B**,**D**). Negative changes indicate fat mass reduction; negative differences favor the extract. *p*-values are from unadjusted between-group comparisons in (**A**) and ANCOVA in (**B**,**D**). ITT analyses used 20 multiply imputed datasets, with inferential estimates pooled using Rubin’s rules. DXA, dual-energy X-ray absorptiometry; PP, per-protocol; ITT, intention-to-treat; SD, standard deviation; CI, confidence interval; IQR, interquartile range; ANCOVA, analysis of covariance.

Efficacy is reported primarily for the PP population, with the ITT population examined for agreement. In the PP population, DXA-measured body fat mass changed by −0.58 ± 1.03 kg in the *L. salicaria* extract group and +1.43 ± 1.22 kg in the control group from baseline to day 84. The corresponding ITT changes were −0.61 ± 1.41 kg and +1.42 ± 1.53 kg, respectively. Comparisons of body fat mass change between the extract and control groups yielded *p* < 0.001 in both the PP and ITT populations (Table 2, Figure 2A). After adjustment for baseline body fat mass, the extract–control difference in change was −2.01 kg (95% CI, −2.51 to −1.52) in the PP population and −2.04 kg (95% CI, −2.66 to −1.42) in the ITT population (*p* < 0.001 for each; Table 2, Figure 2B).

Individual observed changes in body fat mass among the 84 participants with paired baseline and week-12 DXA measurements are shown in Figure 2C. Fat mass decreased in 29 of 42 participants (69.0%) in the extract group and 1 of 42 participants (2.4%) in the placebo group. Median changes were −0.36 kg (interquartile range, −0.94 to 0.17) and +1.12 kg (interquartile range, 0.73 to 1.53), respectively.

#### 3.3.2. Additional and Sensitivity Analyses of the Primary Endpoint

In an additional analysis adjusting for both baseline body fat mass and age, the between-group difference in change in the PP population was −1.93 kg (95% CI, −2.43 to −1.43; *p* < 0.001; Table 2). In the post hoc sensitivity analysis, the percentage changes used to impute missing day-84 body fat mass values were −2.43% in the control group and +2.71% in the extract group. Mean body fat mass changes were +1.29 ± 1.29 kg and −0.49 ± 1.05 kg, respectively. The between-group comparison remained statistically significant using both the independent-samples *t* test and the Mann–Whitney U test (both *p* < 0.001; Table S3).

Two extreme body-fat-mass changes were identified in the placebo group and none in the extract group. After excluding these two participants, the PP sensitivity analysis included 82 participants (40 placebo and 42 extract). The baseline-adjusted extract–control difference was −1.79 kg (95% CI, −2.18 to −1.40; *p* < 0.001), compared with −2.01 kg (95% CI, −2.51 to −1.52; *p* < 0.001) in the original PP analysis (Table 2).

### 3.4. Secondary Efficacy Endpoints

#### 3.4.1. Body Composition and Anthropometry

In the PP population, changes from baseline to day 84 in BMI, body weight, and DXA body fat percentage differed significantly between the groups. BMI was reduced by 0.36 ± 0.56 kg/m^2^ in the *L. salicaria* extract group but increased by 0.26 ± 0.45 kg/m^2^ in the control group (*p* < 0.001). Body weight was reduced by 0.98 ± 1.56 kg versus a 0.79 ± 1.30 kg increase (*p* < 0.001). DXA body fat percentage was reduced by 0.35 ± 0.95 percentage points versus increased by 1.21 ± 0.95 percentage points (*p* < 0.001). For WHR and DXA lean mass, comparisons of baseline-to-day-84 changes in the PP population were nonsignificant (Table 3).

The PP ANCOVA models included each endpoint’s baseline value as a covariate. Estimated differences in day-84 change (extract minus control) were −0.62 kg/m^2^ for BMI (95% CI, −0.84 to −0.40), −1.77 kg for body weight (95% CI, −2.40 to −1.14), and −1.56 percentage points for DXA body fat percentage (95% CI, −1.98 to −1.15), with *p* < 0.001 for each comparison. After baseline adjustment, the comparisons for WHR and DXA lean mass remained nonsignificant (Table 3).

In the ITT analysis, group comparisons of day-84 changes were significant for BMI, body weight, and DXA body fat percentage, but not for WHR or DXA lean mass (Table S4). The unadjusted extract–control differences in change were −2.41 kg (95% CI, −3.29 to −1.52; *p* < 0.001) for body weight and −0.84 kg (95% CI, −1.70 to 0.02; *p* = 0.056) for DXA lean mass.

#### 3.4.2. Blood-Based Metabolic and Inflammatory Markers

PP comparisons of changes from baseline to day 84 were nonsignificant for triglyceride, HDL-C, LDL-C, total cholesterol, FFA, hs-CRP, and leptin (Table 4). In contrast, adiponectin increased by 288 ± 1310 ng/mL in the *L. salicaria* extract group and decreased by 298 ± 1160 ng/mL in the control group (*p* = 0.033).

With baseline adiponectin included as a covariate, the estimated difference in change between the extract and control groups was 605.73 ng/mL (95% CI, 68.34 to 1143.13; *p* = 0.028; Table 4). In the original-scale ANCOVA models, baseline adjustment did not alter the nonsignificant findings for the remaining blood markers.

The ITT findings were consistent: adiponectin changes differed between groups (*p* = 0.031), whereas comparisons for the other blood markers were nonsignificant (Table S4).

#### 3.4.3. Missing-Data Sensitivity Analyses for Body Weight and Lean Mass

Observed baseline measurements and revised imputation summaries for the six participants with missing week-12 body weight and DXA lean mass measurements are presented in Table S5.

In the complete-case analyses using treatment-only linear models with HC3 standard errors, the unadjusted extract–control differences in change were −1.77 kg (95% CI, −2.40 to −1.14; *p* < 0.001) for body weight and −0.24 kg (95% CI, −0.83 to 0.35; *p* = 0.420) for DXA lean mass. Under the adverse missing-data scenarios, the between-group differences were −1.03 kg (95% CI, −1.86 to −0.21; *p* = 0.015) for body weight and −0.73 kg (95% CI, −1.41 to −0.05; *p* = 0.035) for lean mass (Table S6).

In the additional imputation-model analysis using outcome-specific predictive mean matching, unadjusted between-group differences in change were −1.84 kg (95% CI, −2.48 to −1.20) for body weight and −0.32 kg (95% CI, −0.93 to 0.29) for DXA lean mass (Table S6).

#### 3.4.4. Additional Log-Transformed ANCOVA Analyses

In the additional log-transformed ANCOVA, the adjusted extract–control difference in body-weight log change was −0.02 (95% CI, −0.03 to −0.02; *p* < 0.001). Corresponding adjusted differences in log change were −0.11 (95% CI, −0.26 to 0.04; *p* = 0.165) for triglycerides, −0.09 (95% CI, −0.42 to 0.25; *p* = 0.606) for hs-CRP, 0.16 (95% CI, 0.02 to 0.29; *p* = 0.027) for adiponectin, and −0.17 (95% CI, −0.33 to −0.01; *p* = 0.035) for leptin (Table S7).

### 3.5. Dietary Intake, Physical Activity, and Compliance

For changes from baseline to day 84, PP comparisons yielded nonsignificant results for both 3-day energy intake and physical activity. Mean changes in 3-day total energy intake were −233 ± 1074 kcal in the control group and −439 ± 1164 kcal in the *L. salicaria* extract group (between-group *p* = 0.402). Corresponding changes in physical activity were −244 ± 2933 and −37 ± 1935 MET-min/week (MET, metabolic equivalent of task), respectively (between-group *p* = 0.900). Mean compliance was 95.07 ± 4.88% in the control and 97.31 ± 3.60% in the *L. salicaria* extract group; the difference reached significance (*p* = 0.019), although both were above 95% (Table 5).

### 3.6. Safety

Among the 90 participants who took at least one dose, 6 had seven adverse events: three events in 2 *L. salicaria* extract-group participants and four in 4 control-group participants. None were serious, and none led to terminating participation. The *L. salicaria* extract group had one case each of influenza A, migraine, and urticaria; the control group had two common colds, one tonsillitis, and one coronavirus disease. All were mild and unrelated to the product (Table 6). Laboratory tests, vital signs, and examinations showed no clinically significant abnormalities in either group (Table S8).

## 4. Discussion

The results of this trial demonstrated that *L. salicaria* extract reduced body fat compared with placebo in adults with a BMI of 23–30 kg/m^2^. The baseline-adjusted estimates were similar in the PP and ITT populations. The minimal change after additional adjustment for age suggests that the age imbalance in the PP population had limited influence on the estimated between-group effect. However, this analysis does not establish that treatment effects are consistent across age groups or other biological subgroups.

In the PP analysis, body fat mass decreased by 0.58 ± 1.03 kg in the *L. salicaria* extract group and increased by 1.43 ± 1.22 kg in the placebo group over 12 weeks. Body weight showed a similar pattern, decreasing by 0.98 ± 1.56 kg in the extract group and increasing by 0.79 ± 1.30 kg in the placebo group. The absolute reductions in fat mass and body weight within the extract group were modest. The baseline-adjusted between-group differences were −2.01 kg (95% CI, −2.51 to −1.52) for body fat mass and −1.77 kg (95% CI, −2.40 to −1.14) for body weight. The differences between groups reflected opposing changes in fat mass and body weight, which decreased with the extract and increased with the placebo. These increases in the placebo group warrant caution. Their causes could not be determined from the available data. The between-group fat-mass difference was attenuated but remained evident after excluding the two extreme placebo-group observations, suggesting that these observations did not solely account for the primary finding.

Measured changes in dietary intake and physical activity provide context for the body composition results. Comparisons of these changes between groups were nonsignificant. Mean adherence exceeded 95% in both groups. Nevertheless, reliance on participants’ reports for dietary intake and physical activity introduces the possibility of reporting error [23]. Unmeasured changes in eating habits, physical activity, or daily routine cannot be excluded.

A previous clinical study evaluated a combination of *L. salicaria* and *Aceriphyllum rossii*, which limited attribution of findings to *L. salicaria* alone [17]. The present trial adds evidence for an aqueous extract of *L. salicaria* alone, with body fat mass assessed using DXA. However, its efficacy and safety relative to other botanical extracts remain to be established.

DXA enabled direct assessment of changes in body composition. Because neither body weight nor BMI distinguishes fat loss from that of lean tissue [8], changes in DXA-measured fat mass help to interpret the observed weight changes. The accompanying changes in BMI, body weight, and body fat percentage were consistent with the primary fat-mass finding. Individual-level summaries further showed that fat mass decreased in a majority of participants in the extract group, with the median change following the same direction as the mean. Taken together, these findings support a modest short-term reduction in adiposity accompanying the observed weight reduction. This distinction is clinically important because loss of lean mass during weight reduction may impair muscle function [24] and has been associated with subsequent weight regain [25]. Accordingly, interventions that preferentially reduce fat mass while preserving lean tissue are desirable. Between-group differences in lean-mass change were not statistically significant in either the PP or main ITT population. However, the PP confidence interval (−0.82 to 0.36 kg for extract minus placebo) included the possibility of greater lean-mass loss with the extract; therefore, the nonsignificant PP comparison does not establish preservation of lean mass.

Some revised imputed changes in body weight and lean mass remained large and warranted cautious clinical interpretation. Body-weight differences favored the extract across the examined analyses, although the estimated magnitude varied with the missing-data assumptions and imputation specifications. For lean mass, the unadjusted extract–control difference in change was −0.84 kg in the main ITT analysis, compared with −0.32 kg using outcome-specific predictive mean matching. Under the adverse assumptions applied to missing follow-up measurements, the estimated lean-mass difference was −0.73 kg (95% CI, −1.41 to −0.05), indicating greater estimated loss in the extract group under that scenario. Together with the complete-case results, these findings indicate uncertainty in the magnitude of the lean-mass effect and its sensitivity to imputation-model and missing-data assumptions.

The observed reduction in body fat is consistent with previous preclinical observations. In preclinical experiments, *L. salicaria* extract inhibited the differentiation of 3T3-L1 cells into white adipocytes and attenuated body-weight increases associated with high-fat feeding in mice [16]. The proposed mechanisms involved suppression of adipogenesis and lipogenesis, enhanced fatty acid oxidation, and increased AMPK phosphorylation. AMPK responds to changes in cellular energy availability. Its activation suppresses lipid synthesis and promotes catabolic processes that generate energy [26]. The present trial did not include mechanistic measurements; the preclinical results therefore offer hypotheses for the observed reduction in body fat.

The original-scale analyses showed no statistically significant between-group difference in leptin change. The additional log-transformed ANCOVA yielded a nominally significant difference, indicating sensitivity to model specification; this post hoc finding was therefore interpreted as exploratory. Adiponectin changes, however, favored the extract and reached statistical significance in the PP and ITT populations, as well as in the PP analysis adjusted for baseline adiponectin. These hormones have distinct physiological functions. Leptin helps regulate appetite and energy expenditure by signaling the amount of stored energy. Circulating concentrations generally rise with fat mass, but obesity is commonly accompanied by leptin resistance [27]. Greater adiposity and insulin resistance are generally associated with lower circulating adiponectin concentrations [28]. In experimental studies, AMPK and peroxisome proliferator-activated receptor alpha (PPARα) pathways have been implicated in adiponectin-induced increases in fatty acid oxidation and insulin sensitivity [29,30].

However, because multiple secondary endpoints were examined without adjustment for multiple comparisons, the adiponectin finding should be regarded as exploratory. The mechanistic interpretation should also be tentative, because AMPK activity, fatty acid oxidation, markers of adipocyte differentiation, insulin resistance, adiponectin isoforms, and leptin sensitivity were not measured directly, and it remains unclear whether the increase in adiponectin contributes to the reduction in fat or is a consequence of it.

PP comparisons of changes in the lipid panel, FFA, and hs-CRP were nonsignificant. The 12-week duration and the modest magnitude of the change in fat mass may have been insufficient to alter circulating lipids or inflammatory markers. The observed reduction in body fat does not establish an improvement in circulating lipid profiles or a reduction in inflammation.

Concerning safety, all 90 participants who received at least one dose were examined, including 45 who received *L. salicaria* extract. Seven adverse events occurred in six participants; all were mild, and none led to discontinuation. No event was considered causally related to the product, and no clinically significant abnormality was identified in laboratory tests, vital signs, or physical examinations. The *L. salicaria* extract powder, therefore, appears to be well tolerated at 500 mg/day over 12 weeks in individuals comparable to the present participants, although the limited sample size and short duration mean that long-term safety and use in people with coexisting illness remain to be established. These observations provide limited information on uncommon adverse events or safety at other doses.

Nevertheless, some limitations should be noted. Recruitment involved a small sample from a single center, so the findings may not extend to other populations. The lack of directly comparable clinical data introduced uncertainty into the assumed treatment difference and SD used for sample-size planning. Because the principal efficacy analysis used the PP population, selection bias related to post-randomization exclusions cannot be excluded, despite the similar primary-endpoint estimates in the PP and ITT analyses. Diagnostics identified departures from model assumptions in the original-scale ANCOVA analyses. Additional log-transformed analyses and graphical assessments were conducted, although some departures remained, including unequal residual variances for triglycerides. The corresponding adjusted estimates should therefore be interpreted cautiously. The 12-week duration was too short to determine the durability of the fat mass reduction. Comprehensive phytochemical profiling was not performed, and orientin was used only as a marker for extract characterization rather than as an established bioactive compound. The trial was not designed to assess dose–response relationships or mechanistic biomarkers. Glucose was measured as part of the safety assessments and was not a prespecified efficacy endpoint. Glycated hemoglobin (HbA1c) and insulin were not assessed, and the homeostasis model assessment of insulin resistance (HOMA-IR) could not be calculated. This restricted our ability to characterize changes in glycemic control and insulin resistance. The observed changes in body fat and adiponectin therefore do not establish improvements in insulin sensitivity, and their metabolic significance requires further evaluation.

## 5. Conclusions

In adults whose BMI was 23–30 kg/m^2^, 500 mg/day of *L. salicaria* extract powder (corresponding to approximately 400 mg/day of plant-derived extract solids) reduced DXA-assessed body fat mass over 12 weeks compared with placebo. Exploratory secondary analyses also showed between-group differences in BMI, body weight, body fat percentage, and adiponectin. No adverse event was classified as serious; each was mild and judged unrelated to the product. To assess reproducibility, future trials should recruit more participants across multiple centers and extend the follow-up period. Further work is also needed to identify the optimal dose, establish long-term safety, and clarify the mechanism of action.

## Figures and Tables

**Table 1:** Baseline demographic characteristics.

	ITT—Control (*n* = 45)	ITT—Exp (*n* = 45)	*p*	PP—Control (*n* = 42)	PP—Exp (*n* = 42)	*p*
Age, years	36.98 ± 11.31	41.36 ± 12.03	0.079	36.76 ± 11.04	42.05 ± 11.89	0.038
Height, cm	167.30 ± 9.09	166.04 ± 9.01	0.508	167.67 ± 9.10	165.58 ± 9.13	0.297
Weight, kg	71.75 ± 10.25	71.11 ± 7.89	0.742	72.29 ± 10.30	70.40 ± 7.57	0.342
Temperature, °C	36.54 ± 0.30	36.50 ± 0.26	0.554	36.55 ± 0.31	36.51 ± 0.26	0.565
SBP, mmHg	129.20 ± 12.19	129.04 ± 10.94	0.949	129.79 ± 12.01	129.10 ± 10.90	0.783
DBP, mmHg	79.33 ± 10.44	80.11 ± 8.43	0.698	79.24 ± 10.48	80.29 ± 8.36	0.614
Pulse,/min	76.80 ± 9.39	77.24 ± 10.43	0.832	76.19 ± 9.17	77.60 ± 10.65	0.519
Sex, *p*			0.523			0.186
Male	21 (46.7%)	18 (40.0%)		21 (50.0%)	15 (35.7%)	
Female	24 (53.3%)	27 (60.0%)		21 (50.0%)	27 (64.3%)	
Smoking, *p*			0.833			0.458
Non-smoker	37 (82.2%)	38 (84.4%)		34 (81.0%)	36 (85.7%)	
Former	4 (8.9%)	2 (4.4%)		4 (9.5%)	1 (2.4%)	
Current	4 (8.9%)	5 (11.1%)		4 (9.5%)	5 (11.9%)	
Drinking, *p*			0.809			1.000
Non-drinker	12 (26.7%)	11 (24.4%)		10 (23.8%)	10 (23.8%)	
Moderate	33 (73.3%)	34 (75.6%)		32 (76.2%)	32 (76.2%)	
Heavy	0 (0.0%)	0 (0.0%)		0 (0.0%)	0 (0.0%)	

Data are means ± SD or *n* (%). Between-group comparisons of continuous variables used independent-samples *t* tests, with Welch’s *t* test applied when the equal-variance assumption was not met. Categorical variables were compared using the chi-square test or Fisher’s exact test. SD, standard deviation; Exp, experimental group; ITT, intention-to-treat; PP, per-protocol; SBP/DBP, systolic/diastolic blood pressure.

**Table 2:** Changes in DXA-measured body fat mass (kg) in the PP and ITT populations.

Measurement or Analysis	Control	Experimental	Unadjusted Between-Group *p*	Adjusted Difference, kg (95% CI)	ANCOVA *p*
PP, *n* = 42/group					
Baseline	26.29 ± 4.04	25.71 ± 4.49	0.533		
Day 84	27.72 ± 4.35	25.13 ± 4.33	0.008		
Δ to day 84	1.43 ± 1.22	−0.58 ± 1.03	<0.001	−2.01 (−2.51 to −1.52)	<0.001
Median change, kg (IQR)	1.12 (0.73 to 1.53)	−0.36 (−0.94 to 0.17)			
Participants with any decrease, *n* (%)	1 (2.4)	29 (69.0)			
ITT, *n* = 45/group					
Baseline	26.24 ± 3.92	25.67 ± 4.35	0.518		
Day 84	27.65 ± 4.32	25.06 ± 4.29	0.004		
Δ to day 84	1.42 ± 1.53	−0.61 ± 1.41	<0.001	−2.04 (−2.66 to −1.42)	<0.001
Additional PP analyses					
Baseline- and age-adjusted	*n* = 42	*n* = 42		−1.93 (−2.43 to −1.43)	<0.001
Baseline-adjusted analysis excluding extreme values	*n* = 40	*n* = 42		−1.79 (−2.18 to −1.40)	<0.001

Data are mean ± SD unless otherwise indicated; Δ denotes change from baseline. Median changes and proportions with any decrease use observed paired PP data. Any decrease (<0 kg) is descriptive, not a clinically defined response. Unadjusted between-group *p* values are from independent *t* tests. Adjusted differences in change (extract minus control; 95% CI) are from ANCOVA adjusted for baseline body fat mass; the age-adjusted analysis additionally included age. The extreme value analysis excluded two control-group participants with changes outside the group-specific 3-IQR fences. Group-specific sample sizes are shown for the additional PP analyses. ITT analyses used 20 multiply imputed datasets, with inferential estimates pooled using Rubin’s rules. PP, per-protocol; ITT, intention-to-treat; DXA, dual-energy X-ray absorptiometry; SD, standard deviation; IQR, interquartile range; CI, confidence interval; ANCOVA, analysis of covariance.

**Table 3:** Body composition and anthropometric endpoints, baseline to week 12 (PP population).

Variable	Control Mean ± SD	Experimental Mean ± SD	Between Group *p*	Adjusted Difference (95% CI)	ANCOVA *p*
DXA body fat, %					
Baseline	37.30 ± 6.19	37.54 ± 7.17	0.871		
Day 84	38.51 ± 6.09	37.19 ± 7.35	0.371		
Δ to day 84	1.21 ± 0.95	−0.35 ± 0.95	<0.001	−1.56 (−1.98 to −1.15)	<0.001
DXA lean mass, kg					
Baseline	45.07 ± 9.47	43.51 ± 8.65	0.433		
Day 84	45.03 ± 9.66	43.23 ± 8.78	0.374		
Δ to day 84	−0.03 ± 1.41	−0.28 ± 1.28	0.414	−0.23 (−0.82 to 0.36)	0.439
BMI, kg/m^2^					
Baseline	25.67 ± 1.84	25.65 ± 1.54	0.961		
Day 84	25.93 ± 1.98	25.29 ± 1.54	0.106		
Δ to day 84	0.26 ± 0.45	−0.36 ± 0.56	<0.001	−0.62 (−0.84 to −0.40)	<0.001
Weight, kg					
Baseline	72.52 ± 10.47	70.30 ± 7.79	0.273		
Day 84	73.31 ± 10.86	69.32 ± 7.52	0.054		
Δ to day 84	0.79 ± 1.30	−0.98 ± 1.56	<0.001	−1.77 (−2.40 to −1.14)	<0.001
WHR					
Baseline	0.85 ± 0.07	0.84 ± 0.07	0.537		
Day 84	0.85 ± 0.07	0.83 ± 0.06	0.170		
Δ to day 84	0.00 ± 0.05	−0.01 ± 0.05	0.392	−0.01 (−0.03 to 0.01)	0.184

Data are mean ± SD (*n* = 42 per group); Δ denotes change from baseline. Unadjusted between-group *p* values are from independent *t* tests. Adjusted differences in day-84 change (extract—control; 95% CI) are from ANCOVA adjusted for the corresponding baseline value. Body fat percentage differences are expressed in percentage points. *p*-values are not adjusted for multiple comparisons. BMI, body mass index; WHR, waist-to-hip ratio; PP, per-protocol; DXA, dual-energy X-ray absorptiometry; SD, standard deviation; CI, confidence interval; ANCOVA, analysis of covariance.

**Table 4:** Blood-based metabolic and inflammatory markers, baseline to week 12 (PP population).

Variable	Control Mean ± SD	Experimental Mean ± SD	Between Group *p*	Adjusted Difference (95% CI)	ANCOVA *p*
Triglyceride, mg/dL					
Baseline	123.24 ± 114.33	98.76 ± 36.06	0.189		
Day 84	126.07 ± 66.02	101.48 ± 40.37	0.043		
Δ to day 84	2.83 ± 112.11	2.71 ± 31.90	0.112 ^†^	−18.83 (−41.32 to 3.67)	0.100
HDL-C, mg/dL					
Baseline	60.88 ± 13.28	60.69 ± 13.70	0.949		
Day 84	61.71 ± 13.14	59.64 ± 14.97	0.502	—	—
Δ to day 84	0.83 ± 8.76	−1.05 ± 7.04	0.281	−1.91 (−5.29 to 1.48)	0.266
LDL-C, mg/dL					
Baseline	124.69 ± 39.96	121.64 ± 29.44	0.692		
Day 84	119.07 ± 36.59	116.26 ± 31.74	0.708		
Δ to day 84	−5.62 ± 18.01	−5.38 ± 20.70	0.955	−0.30 (−8.34 to 7.74)	0.941
Total cholesterol, mg/dL					
Baseline	198.26 ± 40.66	191.21 ± 32.34	0.382		
Day 84	191.69 ± 40.78	187.31 ± 35.13	0.599		
Δ to day 84	−6.57 ± 29.49	−3.90 ± 23.32	0.785 ^†^	1.08 (−10.01 to 12.17)	0.847
FFA, μEq/L					
Baseline	466.93 ± 169.58	538.17 ± 225.33	0.106		
Day 84	471.95 ± 191.59	542.88 ± 258.22	0.157		
Δ to day 84	5.02 ± 207.61	4.71 ± 254.01	0.995	37.21 (−54.61 to 129.03)	0.422
hs-CRP, mg/L					
Baseline	0.71 ± 0.65	0.98 ± 1.11	0.168		
Day 84	0.88 ± 1.07	1.00 ± 1.35	0.650		
Δ to day 84	0.17 ± 1.20	0.02 ± 1.56	0.127 ^†^	0.06 (−0.47 to 0.59)	0.826
Adiponectin, ng/mL					
Baseline	4066 ± 1778	4386 ± 2988	0.553		
Day 84	3768 ± 1868	4674 ± 3183	0.117		
Δ to day 84	−298 ± 1160	288 ± 1310	0.033	605.73 (68.34 to 1143.13)	0.028
Leptin, pg/mL					
Baseline	12,246 ± 8162	11,518 ± 8369	0.688		
Day 84	12,114 ± 7017	9964 ± 7466	0.178		
Δ to day 84	−132 ± 4066	−1554 ± 5165	0.165	−1621.71 (−3396.48 to 153.05)	0.073

Data are mean ± SD; Δ denotes change from baseline. Unadjusted between-group *p* values are from independent-samples *t* tests unless marked ^†^, which indicates the Mann–Whitney U test. Adjusted differences in day-84 change (extract—control; 95% CI) are from ANCOVA adjusted for the corresponding baseline value. *p*-values are not adjusted for multiple comparisons. FFA, free fatty acids; HDL-C/LDL-C, high-/low-density lipoprotein cholesterol; hs-CRP, high-sensitivity C-reactive protein; PP, per-protocol; SD, standard deviation; CI, confidence interval; ANCOVA, analysis of covariance.

**Table 5:** Dietary intake, physical activity, and compliance during the study (PP population).

Variable	Control (*n* = 42)	Experimental (*n* = 42)	*p* *
Energy intake, 3-day, kcal			
Baseline	4496 ± 1163	4448 ± 1083	0.843
Day 84	4263 ± 1353	4008 ± 1042	0.337
Δ to day 84	−233 ± 1074	−439 ± 1164	0.402
Physical activity, MET-min/wk			
Baseline	2454 ± 4311	1982 ± 1815	0.515
Day 84	2210 ± 2150	1945 ± 1968	0.558
Δ to day 84	−244 ± 2933	−37 ± 1935	0.900 ^†^
Compliance, %			
Intervention	95.07 ± 4.88	97.31 ± 3.60	0.019

Data are mean ± SD; Δ denotes change from baseline to day 84. *p* *, between-group comparison (independent *t*-test unless marked ^†^). ^†^ Mann-Whitney U test. PP, per-protocol; SD, standard deviation; MET, metabolic equivalent of task.

**Table 6:** Summary of adverse events during the 12-week intervention in the safety population.

Variable	Control (*n* = 45)	Experimental (*n* = 45)
Participants with at least one adverse event	4 (8.9)	2 (4.4)
Total number of adverse events	4	3
Adverse events by type		
Common cold	2 (4.4)	0 (0.0)
Coronavirus disease	1 (2.2)	0 (0.0)
Influenza A	0 (0.0)	1 (2.2)
Migraine	0 (0.0)	1 (2.2)
Urticaria	0 (0.0)	1 (2.2)
Tonsillitis	1 (2.2)	0 (0.0)
Participants with serious adverse events	0 (0.0)	0 (0.0)
Participants discontinuing due to adverse events	0 (0.0)	0 (0.0)

Data indicate participant counts and percentages, apart from the row reporting the total event count. A participant could contribute to more than one event category. Each event was classified as mild and judged unrelated to the study product.

## Data Availability

The data presented in this study are available on request from the corresponding author. The data are not publicly available because they contain information related to human participants and are subject to ethical and privacy restrictions.

## Supplementary Materials

The following supporting information can be downloaded at https://www.mdpi.com/article/10.3390/nu18203342/s1, Table S1: Quality-control results for heavy metals and pesticide residues in the *L. salicaria* extract powder; Table S2: Distributional diagnostics and selection of tests for between-group comparisons in the PP and ITT populations; Table S3: Post hoc sensitivity analysis of changes in DXA-measured body fat mass under missing-value assumptions unfavorable to the extract group; Table S4: Changes in secondary efficacy endpoints (ITT set); Table S5: Observed baseline measurements and revised imputation summaries for the six participants with missing week-12 body-weight and DXA lean-mass measurements; Table S6: Comparison of body-weight and DXA lean-mass changes across complete-case, main ITT, and post hoc sensitivity analyses; Table S7: Post hoc ANCOVA analyses of changes in natural-log-transformed outcomes in the PP population; Table S8: Laboratory safety parameters in the safety population; Figure S1: Diagnostic plots for the additional ANCOVA analyses of log-transformed outcomes in the PP population.

## Abbreviations

The following abbreviations are used in this manuscript:

AMPK	AMP-activated protein kinase
ANCOVA	Analysis of covariance
BMI	Body mass index
CI	Confidence interval
CRIS	Clinical Research Information Service
CT	Computed tomography
DXA	Dual-energy X-ray absorptiometry
FFA	Free fatty acid
HDL-C	High-density lipoprotein cholesterol
hs-CRP	High-sensitivity C-reactive protein
IPAQ	International Physical Activity Questionnaire
IRB	Institutional review board
ITT	Intention-to-treat
LDL-C	Low-density lipoprotein cholesterol
LOQ	Limit of quantification
MET	Metabolic equivalent of task
PP	Per-protocol
PPARα	Peroxisome proliferator-activated receptor alpha
SD	Standard deviation
WHO	World Health Organization
WHR	Waist-to-hip ratio

## References

[1] Rubino F., Cummings D.E., Eckel R.H., Cohen R.V., Wilding J.P.H., Brown W.A., Stanford F.C., Batterham R.L., Farooqi I.S., Farpour-Lambert N.J. (2025). Definition and diagnostic criteria of clinical obesity. Lancet Diabetes Endocrinol..

[2] Powell-Wiley T.M., Poirier P., Burke L.E., Després J.P., Gordon-Larsen P., Lavie C.J., Lear S.A., Ndumele C.E., Neeland I.J., Sanders P. (2021). Obesity and cardiovascular disease: A scientific statement from the American Heart Association. Circulation.

[3] World Health Organization (2000). Obesity: Preventing and managing the global epidemic. Report of a WHO consultation. World Health Organ Tech Rep. Ser..

[4] WHO Expert Consultation (2004). Appropriate body-mass index for Asian populations and its implications for policy and intervention strategies. Lancet.

[5] Haam J.H., Kim B.T., Kim E.M., Kwon H., Kang J.H., Park J.H., Kim K.K., Rhee S.Y., Kim Y.H., Lee K.Y. (2023). Diagnosis of obesity: 2022 update of clinical practice guidelines for obesity by the Korean Society for the Study of Obesity. J. Obes. Metab. Syndr..

[6] GBD 2021 Adult BMI Collaborators (2025). Global, regional, and national prevalence of adult overweight and obesity, 1990–2021, with forecasts to 2050: A forecasting study for the Global Burden of Disease Study 2021. Lancet.

[7] Cho S., Jung J.H., Nam G.E., Cho I.Y., Park K.Y., Jeong S.M., Han K. (2025). Taskforce Team of the Obesity Fact Sheet of the Korean Society for the Study of Obesity. 2024 Obesity Fact Sheet in Korea: Prevalence of obesity, abdominal obesity, obesity defined by body fat percentage, and underweight in adults in Korea from 2013 to 2022. J. Obes. Metab. Syndr..

[8] Prentice A.M., Jebb S.A. (2001). Beyond body mass index. Obes. Rev..

[9] Messina C., Albano D., Gitto S., Tofanelli L., Bazzocchi A., Ulivieri F.M., Guglielmi G., Sconfienza L.M. (2020). Body composition with dual energy X-ray absorptiometry: From basics to new tools. Quant. Imaging Med. Surg..

[10] Wharton S., Lau D.C.W., Vallis M., Sharma A.M., Biertho L., Campbell-Scherer D., Adamo K., Alberga A., Bell R., Boulé N. (2020). Obesity in adults: A clinical practice guideline. CMAJ.

[11] Shaik Mohamed Sayed U.F., Moshawih S., Goh H.P., Kifli N., Gupta G., Singh S.K., Chellappan D.K., Dua K., Hermansyah A., Ser H.L. (2023). Natural products as novel anti-obesity agents: Insights into mechanisms of action and potential for therapeutic management. Front Pharmacol..

[12] Wang S., Moustaid-Moussa N., Chen L., Mo H., Shastri A., Su R., Bapat P., Kwun I., Shen C.L. (2014). Novel insights of dietary polyphenols and obesity. J. Nutr. Biochem.

[13] Piwowarski J.P., Granica S., Kiss A.K. (2015). *Lythrum salicaria* L.: Underestimated medicinal plant from European traditional medicine. A review. J. Ethnopharmacol..

[14] Espín J.C., Larrosa M., García-Conesa M.T., Tomás-Barberán F. (2013). Biological significance of urolithins, the gut microbial ellagic acid-derived metabolites: The evidence so far. Evid.-Based Complement. Altern. Med..

[15] Gangwon-do Agricultural Research and Extension Services Selection of Native Wild Vegetables in Gangwon-do for Obesity Prevention. https://www.ares.gangwon.kr/gwares/know_how/research/application_data?articleSeq=31064.

[16] Park Y.J., Kim H.Y., Shin S., Lee J.H., Heo I., Cha Y.Y., An H.J. (2023). Anti-obesity effect of *Lythri herba* water extracts in vitro and in vivo. J. Ethnopharmacol..

[17] Lee D.E., Park W.H., Cha Y.Y. (2012). The effect of the ethanol extracts from *Lythrum salicaria* L. and *Aceriphyllum rossii* on the body composition and temperature of overweight and obese adults: A randomized, double blinded, placebo food controlled, single center, preliminary human study. J. Korean Med. Rehabil..

[18] Hopewell S., Chan A.W., Collins G.S., Hróbjartsson A., Moher D., Schulz K.F., Tunn R., Aggarwal R., Berkwits M., Berlin J.A. (2025). CONSORT 2025 Statement: Updated guideline for reporting randomised trials. BMJ.

[19] Park H.J., Heo I., Park Y.J., An H.J., Shin S., Cha Y.Y. (2024). Effect of *Lythrum salicaria* extract on body fat reduction: A protocol for a randomized, double-blinded, placebo-controlled clinical trial. J. Korean Med. Obes. Res..

[20] Reagan-Shaw S., Nihal M., Ahmad N. (2008). Dose translation from animal to human studies revisited. FASEB J..

[21] Craig C.L., Marshall A.L., Sjöström M., Bauman A.E., Booth M.L., Ainsworth B.E., Pratt M., Ekelund U., Yngve A., Sallis J.F. (2003). International Physical Activity Questionnaire: 12-country reliability and validity. Med. Sci. Sports Exerc.

[22] Sterne J.A.C., White I.R., Carlin J.B., Spratt M., Royston P., Kenward M.G., Wood A.M., Carpenter J.R. (2009). Multiple imputation for missing data in epidemiological and clinical research: Potential and pitfalls. BMJ.

[23] Lichtman S.W., Pisarska K., Berman E.R., Pestone M., Dowling H., Offenbacher E., Weisel H., Heshka S., Matthews D.E., Heymsfield S.B. (1992). Discrepancy between self-reported and actual caloric intake and exercise in obese subjects. N. Engl. J. Med..

[24] Weiss E.P., Racette S.B., Villareal D.T., Fontana L., Steger-May K., Schechtman K.B., Klein S., Ehsani A.A., Holloszy J.O., Washington University School of Medicine CALERIE Group (2007). Lower extremity muscle size and strength and aerobic capacity decrease with caloric restriction but not with exercise-induced weight loss. J. Appl. Physiol..

[25] Martins C., Gower B.A., Hunter G.R. (2022). Association between Fat-Free Mass Loss after Diet and Exercise Interventions and Weight Regain in Women with Overweight. Med. Sci. Sports Exerc..

[26] Hardie D.G., Ross F.A., Hawley S.A. (2012). AMPK: A nutrient and energy sensor that maintains energy homeostasis. Nat. Rev. Mol. Cell Biol..

[27] Obradovic M., Sudar-Milovanovic E., Soskic S., Essack M., Arya S., Stewart A.J., Gojobori T., Isenovic E.R. (2021). Leptin and obesity: Role and clinical implication. Front Endocrinol..

[28] Kern P.A., Di Gregorio G.B., Lu T., Rassouli N., Ranganathan G. (2003). Adiponectin expression from human adipose tissue: Relation to obesity, insulin resistance, and tumor necrosis factor-alpha expression. Diabetes.

[29] Kadowaki T., Yamauchi T. (2005). Adiponectin and adiponectin receptors. Endocr. Rev..

[30] Yamauchi T., Kamon J., Minokoshi Y., Ito Y., Waki H., Uchida S., Yamashita S., Noda M., Kita S., Ueki K. (2002). Adiponectin stimulates glucose utilization and fatty-acid oxidation by activating AMP-activated protein kinase. Nat. Med..

