# Supplementary material for: Efficacy and Safety of Lythrum salicaria Extract on Body Fat Reduction in Adults with a BMI of 23–30 kg/m2: A Randomized, Double-Blind, Placebo-Controlled Trial
Source: Nutrients. 2026 Oct 12;18(20):3342. doi: 10.3390/nu18203342 (PMC13708396; doi:10.3390/nu18203342)
Supplement: Supplementary file 1 [file nutrients-18-03342-s001.zip › nutrients-4552952-supplementary.pdf]

nutrients

MDPI

Supplementary Materials

Efficacy and Safety of *Lythrum salicaria* Extract on Body Fat Reduction in Adults with a BMI of 23–30 kg/m^2: A Randomized, Double-Blind, Placebo-Controlled Trial

Bong-Jin Shin [1,2], Hye-Jin Park [3], Yea-Jin Park [4,5], Hyo-Jin An [5,6], Su Shin [7], Yun-Yeop Cha [4,*] and In Heo [2,8,*]

1 Department of Korean Medicine, The Graduate School, Pusan National University, Yangsan 50612, Republic of Korea; shinbj0831@gmail.com
2 Department of Korean Medicine Rehabilitation, Pusan National University Korean Medicine Hospital, 20 Geumo-ro, Mulgeum-eup, Yangsan 50612, Republic of Korea
3 Department of Rehabilitation Medicine of Korean Medicine, Se-Myung University Chungju Korean Medicine Hospital, 63 Sangbang 4-gil, Chungju 27429, Republic of Korea
4 Department of Rehabilitation Medicine of Korean Medicine, College of Korean Medicine, Sangji University, 80 Sangjidae-gil, Wonju 26338, Republic of Korea
5 College of Pharmacy and Institute of Integrated Pharmaceutical Sciences, Kyung Hee University, 26 Kyungheedae-ro, Dongdaemun-gu, Seoul 02447, Republic of Korea
6 Department of Precision Medicine, Graduate School, Kyung Hee University, Seoul 02447, Republic of Korea
7 Department of Korean Medical Science, School of Korean Medicine, Pusan National University, Yangsan 50612, Republic of Korea
8 The School of Korean Medicine, Pusan National University, Yangsan 50612, Republic of Korea
* Correspondence: omdcha@sangji.ac.kr (Y.-Y.C.); drheoin@pusan.ac.kr (I.H.); Tel.: +82-33-730-01-260 (Y.-Y.C.); +82-55-360-5966 (I.H.)

Table S1. Quality-control results for heavy metals and pesticide residues in the *L. salicaria* extract powder.

Variable	Result (mg/kg)	LOQ (mg/kg)
**Lead**	0.17	-
**Cadmium**	0.05	-
**Arsenic**	0.37-0.39	-
**Total mercury**	0.00	-
**Pesticide panel (117 analytes)**<br>**Diazinon; Deltamethrin; Dinotefuran; DDT; Dicofol; Dichlorvos; Diflubenzuron; Lufenuron; Malathion; Methomyl; Methoxyfenozide; Buprofezin; Bifenthrin; Spinosad; Spirodiclofen; Spiromesifen; Cypermethrin; Cyfluthrin; Cyhalothrin; Acetamiprid; Imidacloprid; Isoprocarb; Indoxacarb; Quinalphos; Chlorantraniliprole; Chlorpyrifos; Chlorpyrifos-methyl; Chlorfenapyr; Clothianidin; Tebufenozide; Triflumuron; Thiamethoxam; Thiacloprid; Permethrin; Fenitrothion; Fenvalerate; Phenthoate; Fenpropathrin; Flucythrinate; Prothiofos; Propargite; Profenofos; Flubendiamide; Pyridaben; Pirimiphos-methyl; Pyriproxyfen; Fipronil; Hexaflumuron; Chlorobenzuron; Nitenpyram; Tolfenpyrad; Dimethoate; Methiocarb; Methamidophos; Methidathion; Ethion; Endosulfan; Omethoate; Carbaryl; Carbofuran; Triazophos; Ethofumesate; Diniconazole; Dimethomorph; Difenoconazole; Diphenylamine; Mandipropamid; Metalaxyl; Boscalid; Cymoxanil; Cyprodinil; Cyproconazole; Azoxystrobin; Ortho-phenylphenol; Imazalil; Isoprothiolane; Iprodione; Carbendazim; Carboxin; Quintozene; Chlorothalonil; Tebuconazole; Tolclofos-methyl; Tricyclazole; Triadimenol; Triadimefon; Trifloxystrobin; Thiabendazole; Famoxadone; Procymidone; Prochloraz; Propamocarb; Propiconazole; Pyraclostrobin; Pyrimethanil; Hexaconazole; Fludioxonil; Bupirimate; Metominostrobin; Flutriafol; Diuron; Linuron; Butachlor; Acetochlor; Oxadiazon; Oxyfluorfen; Atrazine; Tri-allate; Phenmedipham; Metamitron; Paclobutrazol;**	ND (<LOQ)	0.01

2,6-Diisopropylnaphthalene (2,6-DIPN); Piperonyl butoxide; Endrin; Dieldrin; Aldrin; BHC.

ND, not detected (<LOQ); LOQ, limit of quantification; DDT, dichlorodiphenyltrichloroethane; BHC, benzene hexachloride; -, LOQ not reported. Results were identical across three lots (F12210001–F12210003), except for arsenic (range shown). Total mercury values reflect rounding. The testing laboratory confirmed an LOQ of 0.01 mg/kg for each of the 117 pesticides.

Table S2. Distributional diagnostics and selection of tests for between-group comparisons in the PP and ITT populations.

Variable	PP population (*n* = 42 per group)						ITT population (*n* = 45 per group)					
	Distributional diagnostics (Control / Extract)				Test selection		Distributional diagnostics (Control / Extract)				Test selection	
	Shapiro–Wilk *p*	Skewness	Kurtosis	EV	Variance *p*	Test	Shapiro–Wilk *p*	Skewness	Kurtosis	EV	Variance *p*	Test
**Age**	0.001 / 0.133	1.217 / 0.457	1.579 / −0.541	−/−	0.260	*t*	0.001 / 0.102	1.183 / 0.450	1.177 / −0.532	−/−	0.319	*t*
**Height**	0.213 / 0.004	−0.345 / 0.665	−0.764 / −0.726	−/−	0.970	*t*	0.150 / 0.010	−0.268 / 0.530	−0.887 / −0.857	−/−	0.968	*t*
**Body weight**	0.207 / 0.150	0.168 / 0.499	−0.998 / −0.537	−/−	0.019	Wt	0.131 / 0.176	0.248 / 0.427	−0.964 / −0.649	−/−	0.040	Wt
**Body temperature**	0.270 / 0.203	0.237 / −0.121	-0.664 / −0.257	−/−	0.161	*t*	0.169 / 0.206	0.301 / −0.118	-0.118 / −0.254	-/−	0.149	*t*
**SBP**	0.409 / 0.172	−0.116 / 0.095	1.205 / −0.979	−/−	0.897	*t*	0.409 / 0.133	−0.092 / 0.079	0.852 / −1.020	−/−	0.919	*t*
**DBP**	0.363 / 0.596	0.107 / 0.172	−0.843 / 0.139	−/−	0.071	*t*	0.278 / 0.934	0.104 / 0.086	−0.881 / 0.071	−/−	0.072	*t*
**Pulse**	0.960 / 0.066	0.221 / 0.753	−0.360 / 1.548	−/−	0.873	*t*	0.942 / 0.038	0.205 / 0.817	−0.447 / 1.727	−/−	0.915	*t*
**DXA body fat mass**	<0.001 / <0.001	2.546 / −1.578	7.398 / 2.790	+/−	0.987	*t*	<0.001 / <0.001	2.463 / -1.341	7.292 / 1.816	+/−	0.757	*t*
**DXA body fat percentage**	0.280 / 0.177	0.551 / −0.680	0.244 / 0.173	−/−	0.778	*t*	0.673 / 0.005	0.313 / 0.934	0.190 / 3.439	−/+	0.561	*t*
**DXA lean mass**	0.312 / 0.371	0.487 / −0.377	0.722 / 0.882	−/−	0.414	*t*	0.049 / <0.001	0.854 / -2.398	1.280 / 9.127	−/+	0.668	*t*

**BMI**	0.205 / 0.048	0.324 / −0.884	−0.634 / 2.694	−/−	0.522	*t*	<0.001 / <0.001	1.728 / -1.758	4.248 / 5.032	+/+	0.519	*t*
**Body weight**	0.056 / 0.001	0.763 / −1.529	0.263 / 5.840	−/−	0.705	*t*	<0.001 / <0.001	1.961 / -2.075	5.214 / 6.366	+/+	0.730	*t*
**WHR**	<0.001 / <0.001	−0.125 / −0.199	1.487 / 0.586	+/+	0.263	*t*	<0.001 / <0.001	0.000 / -0.215	0.987 / 0.500	+/+	0.206	*t*
**Triglyceride**	<0.001 / 0.016	−4.405 / 1.101	24.984 / 2.012	+/−	0.047	*U*	<0.001 / 0.005	-4.545 / 1.195	26.657 / 2.184	+/−	0.049	*Rt*
**HDL-C**	0.005 / 0.052	1.144 / 0.851	3.937 / 1.134	+/−	0.616	*t*	0.013 / 0.042	0.986 / 0.823	3.398 / 0.990	−/−	0.516	*t*
**LDL-C**	0.642 / 0.043	0.475 / −0.618	0.339 / 2.337	−/−	0.668	*t*	0.705 / 0.087	0.393 / -0.529	0.203 / 2.035	−/−	0.607	*t*
**Total cholesterol**	<0.001 / 0.299	−2.498 / −0.377	11.483 / 1.291	+/−	0.840	*U*	<0.001 / 0.241	-2.483 / -0.305	11.435 / 1.216	+/−	0.775	*Rt*
**FFA**	0.211 / 0.487	−0.268 / −0.441	−0.655 / −0.083	−/−	0.402	*t*	0.212 / 0.673	-0.256 / -0.307	-0.592 / -0.171	−/−	0.211	*t*
**hs-CRP**	<0.001 / <0.001	2.385 / 1.813	14.530 / 10.061	+/+	0.366	*U*	<0.001 / <0.001	1.693 / 1.769	11.216 / 10.384	+/+	0.546	*Rt*
**Adiponectin**	0.047 / 0.003	0.899 / 1.177	2.643 / 3.770	−/+	0.767	*t*	0.034 / 0.010	0.899 / 1.008	2.734 / 3.350	−/+	0.616	*t*
**Leptin**	0.023 / 0.007	−0.213 / −0.851	1.004 / 0.613	−/+	0.120	*t*	0.034 / 0.010	-0.261 / -0.883	1.224 / 0.746	+/+	0.091	*t*
**3-day total energy intake**	0.053 / 0.825	1.022 / −0.375	2.113 / −0.082	−/−	0.561	*t*	0.059 / 0.750	0.959 / -0.402	2.183 / 0.067	−/−	0.589	*t*
**Physical activity**	<0.001 / 0.016	−2.924 / −0.608	10.759 / 2.209	+/+	0.523	*U*	<0.001 / 0.008	-3.027 / -0.576	11.619 / 2.458	+/+	0.502	*Rt*
**WBC**	0.285 / <0.001	−0.350 / −1.966	0.722 / 9.317	−/+	0.505	*U*	0.141 / <0.001	-0.465 / -2.004	0.803 / 9.946	−/+	0.584	*Rt*
**Hemoglobin**	0.443 / 0.528	−0.243 / −0.358	−0.313 / −0.322	−/−	0.622	*t*	0.398 / 0.572	-0.324 / -0.304	-0.162 / -0.265	−/−	0.656	*t*
**Platelet count**	0.335 / 0.015	−0.323 / 1.148	0.424 / 2.843	−/−	0.838	*t*	0.227 / 0.011	-0.444 / 1.146	0.481 / 3.079	−/−	0.677	*t*
**AST**	<0.001 / <0.001	−1.357 / −2.001	2.918 / 7.853	+/+	0.706	*t*	<0.001 / <0.001	-1.360 / -2.088	2.941 / 8.409	−/+	0.613	*t*

**ALT**	<0.001 / 0.033	−3.168 / −0.549	13.814 / 1.851	+/+	0.337	*U*	<0.001 / 0.018	-3.227 / -0.624	14.475 / 2.110	+/+	0.613	*Rt*
**Creatinine**	0.338 / 0.178	−0.314 / 0.401	0.936 / -0.385	−/−	0.896	*t*	0.194 / 0.154	-0.412 / 0.420	1.128 / -0.279	−/−	0.883	*t*
**Glucose**	0.802 / 0.097	−0.198 / 0.637	0.460 / 0.137	−/−	0.933	*t*	0.746 / 0.049	-0.158 / 0.678	0.582 / 0.373	−/−	0.806	*t*

Within each population, slash-separated values are control / *L. salicaria* extract. The first seven rows (age through pulse) report baseline values; all others report changes (day 84 minus baseline). Shapiro–Wilk *p* tests within-group normality; variance *p* tests between-group variance homogeneity. EV, boxplot-defined extreme values (+, present; -, absent). Test codes: t, independent-samples t test; Wt, Welch's t test for unequal variances; U, Mann–Whitney U test; Rt, independent-samples t test on ranks calculated separately within each imputed dataset, with mean-rank differences and their variances pooled using Rubin's rules. Test selection considered all diagnostics. ITT diagnostics used a descriptive dataset with missing entries replaced by their means across 20 imputations; these diagnostics were not pooled. ITT inferential analyses were performed within each imputed dataset and pooled separately.

PP, per-protocol; ITT, intention-to-treat; SBP, systolic blood pressure; DBP, diastolic blood pressure; DXA, dual-energy X-ray absorptiometry; BMI, body mass index; WHR, waist-to-hip ratio; HDL-C/LDL-C, high-/low-density lipoprotein cholesterol; FFA, free fatty acids; hs-CRP, high-sensitivity C-reactive protein; WBC, white blood cell count; AST, aspartate aminotransferase; ALT, alanine aminotransferase.

Table S3. Post hoc sensitivity analysis of changes in DXA-measured body fat mass under missing-value assumptions unfavorable to the extract group.

Variable	Control (*n* = 45)	Experimental (*n* = 45)	Independent-samples t test, *p*	Mann–Whitney U test, *p*
**Change in body fat mass from baseline to day 84, kg**	+1.29 ± 1.29	−0.49 ± 1.05	<0.001	<0.001

Data are mean ± SD. Missing day-84 values were imputed using the greatest observed percentage decrease within the control group (−2.43%) and the greatest observed percentage increase within the extract group (+2.71%). *p* values compare changes between groups. DXA, dual-energy X-ray absorptiometry; SD, standard deviation.

Table S4. Changes in secondary efficacy endpoints (ITT set).

Variable	Control (*n* = 45)	Experimental (*n* = 45)	*p**
Body composition			
DXA body fat, %			
Baseline	37.54±6.09	37.14±7.15	0.771
Day 84	38.64±5.98	36.98±7.19	0.235
Δ to day 84	1.09±1.29	-0.16±1.44	<0.001
DXA lean mass, kg			
Baseline	44.53±9.44	44.29±9.00	0.899
Day 84	44.69±9.47	43.60±8.69	0.571
Δ to day 84	0.15±1.77	−0.69±2.30	0.056
Anthropometric outcomes			
BMI, kg/m^2			
Baseline	25.57±1.82	25.76±1.56	0.603
Day 84	25.93±1.98	25.27±1.51	0.075
Δ to day 84	0.36±0.70	−0.49±0.81	<0.001
Weight, kg			
Baseline	71.94±10.43	71.02±8.10	0.642
Day 84	73.01±10.59	69.68±7.44	0.085
Δ to day 84	1.07±2.05	−1.34±2.27	<0.001
WHR			
Baseline	0.84±0.08	0.84±0.07	0.884
Day 84	0.84±0.07	0.83±0.06	0.219
Δ to day 84	0.00±0.05	−0.01±0.05	0.208
Blood-based metabolic and inflammatory markers			
Triglyceride, mg/dL			
Baseline	122.22±111.25	100.44±35.48	0.211

Day 84	126.50±69.87	101.43±42.61	0.040
Δ to day 84	4.28±111.99	0.98±35.78	0.078†
HDL-C, mg/dL			
Baseline	60.67±13.04	60.49±13.45	0.949
Day 84	61.98±13.52	59.47±15.13	0.411
Δ to day 84	1.31±9.74	-1.01±8.24	0.230
LDL-C, mg/dL			
Baseline	123.53±38.99	122.31±29.36	0.867
Day 84	118.71±37.23	116.22±33.00	0.740
Δ to day 84	-4.82±21.27	-6.09±23.38	0.795
Total cholesterol, mg/dL			
Baseline	196.93±39.76	192.20±32.30	0.535
Day 84	192.07±41.48	187.63±37.93	0.603
Δ to day 84	-4.87±31.52	-4.57±27.81	0.932†
FFA, μEq/L			
Baseline	467.53±172.56	535.56±229.80	0.112
Day 84	472.23±210.08	547.54±279.05	0.160
Δ to day 84	4.69±223.05	11.99±288.48	0.896
hs-CRP, mg/L			
Baseline	0.78±0.77	0.95±1.08	0.396
Day 84	0.94±1.31	1.01±1.43	0.812
Δ to day 84	0.15±1.47	0.06±1.62	0.228†
Adiponectin, ng/mL			
Baseline	4107±1801	4285±2951	0.729
Day 84	3789±1928	4644±3277	0.133
Δ to day 84	-318±1267	359±1629	0.031
Leptin, pg/mL			
Baseline	12,550±8072	11,086±8245	0.395
Day 84	12,481±7379	9640±7533	0.073
Δ to day 84	-69±4446	-1446±5341	0.192

Data are mean ± SD; Δ denotes change from baseline. *p**, between-group comparison (independent t test unless marked †). † Pooled results from independent-samples t tests on values rank-transformed separately within each of the 20 imputed datasets; mean-rank differences and their variances were combined using Rubin's rules. Missing values were multiply imputed. All *p* values are nominal, without adjustment for multiple comparisons. ITT, intention-to-treat; DXA, dual-energy X-ray absorptiometry; BMI, body mass index; WHR, waist-to-hip ratio; FFA, free fatty acids; HDL-C/LDL-C, high-/low-density lipoprotein cholesterol; hs-CRP, high-sensitivity C-reactive protein; SD, standard deviation.

Table S5. Observed baseline measurements and revised imputation summaries for the six participants with missing week-12 body-weight and DXA lean-mass measurements.

**A. Individual baseline measurements and mean imputed week-12 values**					
**Participant**	Group	Body weight: baseline	Body weight: revised week-12 mean	Lean mass: baseline	Lean mass: revised week-12 mean
**002**	Control	70.5	71.0	42.775	42.849
**075**	Control	58.2	66.6	31.349	36.436
**078**	Control	62.6	68.7	37.118	40.228
**041**	Extract	77.5	72.3	48.356	43.970
**043**	Extract	88.0	77.9	63.279	52.961

**082**	Extract	78.0	74.2	54.024	49.368

B. Reported overall ranges of imputed week-12 values and changes from baseline

Outcome	Revised week-12 range	Revised change range
**Body weight**	66.6 to 77.9	−10.1 to +8.4
**DXA lean mass**	36.436 to 52.961	−10.318 to +5.087

All values are in kg. Baseline measurements are observed values. Week-12 entries in panel A are participant-specific means across the 20 imputed datasets, not observed follow-up measurements. Panel B presents the reported overall ranges across the six participants and the corresponding imputed datasets. Change was defined as week 12 minus baseline. DXA, dual-energy X-ray absorptiometry.

Table S6. Comparison of body-weight and DXA lean-mass changes across complete-case, main ITT, and post hoc sensitivity analyses.

Analysis	*n*	Body-weight difference, kg (95% CI)	*p*	Lean-mass difference, kg (95% CI)	*p*
**Complete-case analysis**	84	−1.77 (−2.40 to −1.14)	<0.001	−0.24 (−0.83 to 0.35)	0.420
**Main ITT analysis, 20 imputations**	90	−2.41 (−3.29 to −1.52)	<0.001	−0.84 (−1.70 to 0.02)	0.056
**Adverse missing-data scenario**	90	−1.03 (−1.86 to −0.21)	0.015	−0.73 (−1.41 to −0.05)	0.035
**Outcome-specific PMM, 20 imputations**	90	−1.84 (−2.48 to −1.20)	<0.001	−0.32 (−0.93 to 0.29)	0.296

Effects are unadjusted between-group differences in mean change (extract minus control), expressed in kg with 95% confidence intervals. Change was defined as week 12 minus baseline. Complete-case analyses included 84 participants (42 per group); the remaining analyses included 90 participants (45 per group). Complete-case and outcome-specific PMM analyses used treatment-only linear models with HC3 heteroscedasticity-consistent standard errors. Main ITT and adverse-scenario comparisons used independent-samples t tests. For both multiple-imputation analyses, effect estimates and variances were pooled across 20 imputed datasets using Rubin's rules. The adverse scenarios retained observed week-12 measurements and applied alternative percentage changes to baseline values for participants with missing follow-up data: −8.53% and +4.71% for body weight in the control and extract groups, respectively, and +7.33% and −8.78% for lean mass. These assumptions were based on the extreme observed percentage changes across both groups. All *p* values are nominal, without adjustment for multiple comparisons. DXA, dual-energy X-ray absorptiometry; ITT, intention-to-treat; PMM, predictive mean matching; CI, confidence interval.

Table S7. Post hoc ANCOVA analyses of changes in natural-log-transformed outcomes in the PP population.

Outcome	Adjusted difference in log change (95% CI)	*p*
**Body weight**	−0.02 (−0.03 to −0.02)	<0.001
**Triglycerides**	−0.11 (−0.26 to 0.04)	0.165
**hs-CRP**	−0.09 (−0.42 to 0.25)	0.606
**Adiponectin**	0.16 (0.02 to 0.29)	0.027
**Leptin**	−0.17 (−0.33 to −0.01)	0.035

Effects are adjusted between-group differences (extract minus control) in log change, defined as ln(week-12 value) minus ln(baseline value), in the PP population (n = 42 per group). Estimates and conventional model-based 95% confidence intervals were obtained using ANCOVA with baseline adjustment. p values are nominal and unadjusted for multiple comparisons. PP, per-protocol; ANCOVA, analysis of covariance; CI, confidence interval.

Table S8. Laboratory safety parameters in the safety population.

Variable	Control (n = 45)	Experimental (n = 45)	p*
**WBC, ×10³/μL**			
**Baseline**	6.07±1.47	6.16±1.62	0.766
**Day 84**	5.57±1.30	5.83±1.32	0.333
**Δ to day 84**	-0.50±1.23	-0.33±1.62	0.496†
**Hemoglobin, g/dL**			
**Baseline**	14.16±1.42	13.89±1.44	0.375
**Day 84**	14.09±1.53	13.92±1.44	0.578
**Δ to day 84**	-0.07±0.64	0.03±0.58	0.461
**Platelet, ×10³/μL**			
**Baseline**	281.13±73.63	271.51±62.58	0.504
**Day 84**	266.52±70.47	264.45±61.40	0.882
**Δ to day 84**	-14.62±33.73	-7.06±31.32	0.266
**AST, U/L**			
**Baseline**	22.29±8.11	22.33±7.87	0.979
**Day 84**	22.27±6.32	21.64±6.51	0.640
**Δ to day 84**	-0.02±7.44	-0.69±7.38	0.665
**ALT, U/L**			
**Baseline**	21.73±17.19	19.44±10.31	0.444
**Day 84**	21.03±15.74	18.62±9.31	0.376
**Δ to day 84**	-0.70±12.75	-0.82±8.64	0.219†
**Creatinine, mg/dL**			
**Baseline**	0.78±0.17	0.77±0.18	0.760
**Day 84**	0.77±0.18	0.77±0.17	0.933
**Δ to day 84**	-0.01±0.08	0.00±0.07	0.630
**Glucose, mg/dL**			
**Baseline**	63.49±11.58	64.44±13.82	0.722
**Day 84**	62.52±13.39	58.15±12.40	0.110
**Δ to day 84**	-0.97±14.96	-6.29±14.55	0.088

Data are mean ± SD; Δ denotes change from baseline to day 84. p*, between-group comparison (independent t test unless marked †). † Pooled results from independent-samples t tests on values rank-transformed separately within each of the 20 imputed datasets; mean-rank differences and their variances were combined using Rubin's rules. Missing laboratory values were multiply imputed.

WBC, white blood cell; AST, aspartate aminotransferase; ALT, alanine aminotransferase; SD, standard deviation.

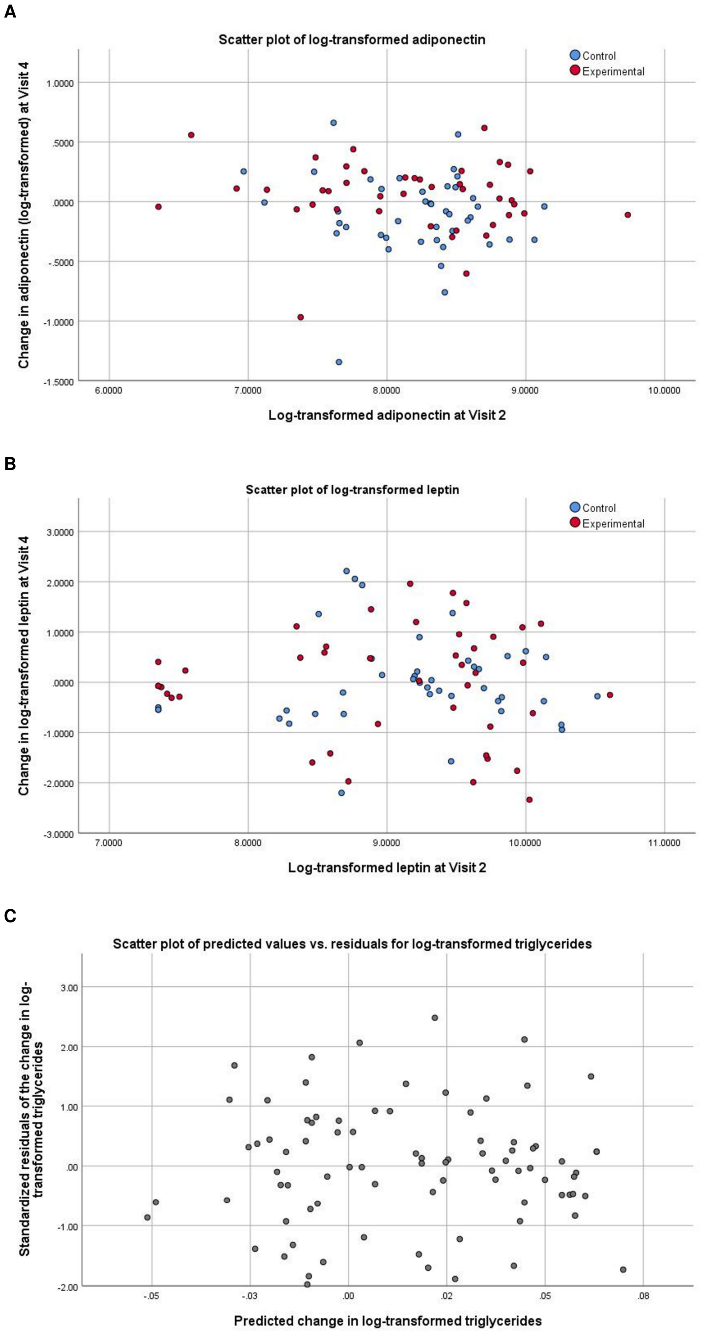

Figure S1. Diagnostic plots for the additional ANCOVA analyses of log-transformed outcomes in the PP population. (A,B) Relationships between the natural logarithm of the baseline measurement (Visit 2) and the change in the log-transformed measurement from baseline to week 12 (Visit 4) for adiponectin (A) and leptin (B). Each point represents one participant; blue and red points denote the control and *L. salicaria* extract (Experimental) groups, respectively. (C) Standardized residuals plotted against predicted changes in log-transformed triglycerides. Log change was defined as ln(week-12 value) minus ln(baseline value). These plots were examined alongside formal diagnostic tests to assess baseline–change relationships and residual variability. PP, per-protocol; ANCOVA, analysis of covariance.
